# Physio-pathological effects of m6A modification and its potential contribution to melanoma

**DOI:** 10.1007/s12094-021-02644-3

**Published:** 2021-06-08

**Authors:** Y. Liao, P. Han, Y. Zhang, B. Ni

**Affiliations:** 1 Department of Oncology, Chongqing General Hospital, University of Chinese Academy of Sciences, Chongqing, 400013 China; 2 Chongqing International Institute of Immunology, Chongqing, 400018 China; 3 Department of Pathophysiology, College of High Altitude Military Medicine, Third Military Medical University, Chongqing, 400038 China

**Keywords:** RNA modification, m6A, Tumor, Melanoma

## Abstract

Methylation of N6-adenosine (m6A) is the most prevalent internal RNA modification and is especially common among the messenger RNAs. These m6A modifications regulate splicing, translocation, stability and translation of RNA through dynamic and reversible interactions with m6A-binding proteins, namely the writers, erasers and readers. RNA methyltransferases catalyze the m6A modifications, while demethylases reverse this methylation. Deregulation of the m6A modification process has been implicated in human carcinogenesis, including melanoma—which carries one of the highest mutant rates. In this review, we provide an up-to-date summary of m6A regulation and its biological impacts on normal and cancer cells, with emphasis on the deregulation of m6A modification and m6A regulators in melanoma. In addition, we highlight the prospective potential of exploiting m6A modification in the treatment of melanoma and non-cancer diseases.

## Introduction

RNA is a critical carrier of genetic information and catalyzes various biochemical reactions by providing vital information for protein synthesis and acting as a scaffold for attaching the amino acids in proper sequential order. Eukaryotic RNA can be modified in various ways, as previously documented [1]. To date, over 100 kinds of RNA modifications have been identified in eukaryotes and among the various classes of RNAs, including the messenger RNAs (mRNAs) [2, 3], transport RNAs(tRNAs) [4], microRNAs (miRNAs) [5, 6], long non-coding RNAs [3, 7], and circular RNAs [8, 9].

Within the various types of RNA modifications, capping at the 5'end and polyA modification at the 3' end play very important roles in transcriptional regulation. The methylation modification of mRNA at the 5' cap is a highly regulated process, being crucial for the creation of mature mRNA and involved in maintenance of the mature mRNA’s stability, its nuclear exportation, and ability to initiate translation [10]. The polyA modifications, which occur via polyA-binding proteins at the 3' end, similarly contribute to nuclear transport, the maintenance of structural stability, and the functional initiation of translation. Major methylation modifications of RNA include N6-methyladenosine (m6A), 5-methylcytosine, N1-methyladenosine, 5-hydroxymethylcytosine, N6, 2’-O-dimethyladenosine, and 7-methylguanine [11–13]. Of these, m6A modifications mainly occur in the RRACH sequence (where R = A or G, and H = A, C, or U) in nature [14] and it is the most prevalent chemical modification identified for eukaryotic mRNAs [6, 15]. Indeed, it has been estimated that approximately 0.1% to 0.4% of adenosines in mRNA are subjected to m6A modification, with, on average, two to three m6A-modified sites per transcript [16].

The m6A modification involves an addition of a methyl group at position N6 of adenosine. Being highly conserved evolutionarily [16], and present in most organisms from bacteria to mammals. m6A is also the most abundant chemical modification found on mammalian mRNA and involved in the regulation of a wide array of cellular processes [17]. Not surprisingly, then, alteration of the m6A modification has been found to contribute to the development of various disease states, including obesity [18], infertility [19], autoimmune disease [20], and neurological disease [21]. Deregulation of m6A modification has also recently been implicated in cancer. Although rapid progress has been made in the study of this newly recognized pathogenic mechanism, they are limited by the fact that our knowledge on the regulatory networks of m6Ais not yet complete. In this review, we summarize the most recent findings on the functions of m6A modification in normal and cancer cells, with particular emphasis on the role of m6A modification in melanoma. Finally, we highlight the potential applications and possible future directions in this burgeoning field that will benefit human health.

## Basic biology of m6A modification

**Fig. 1:**
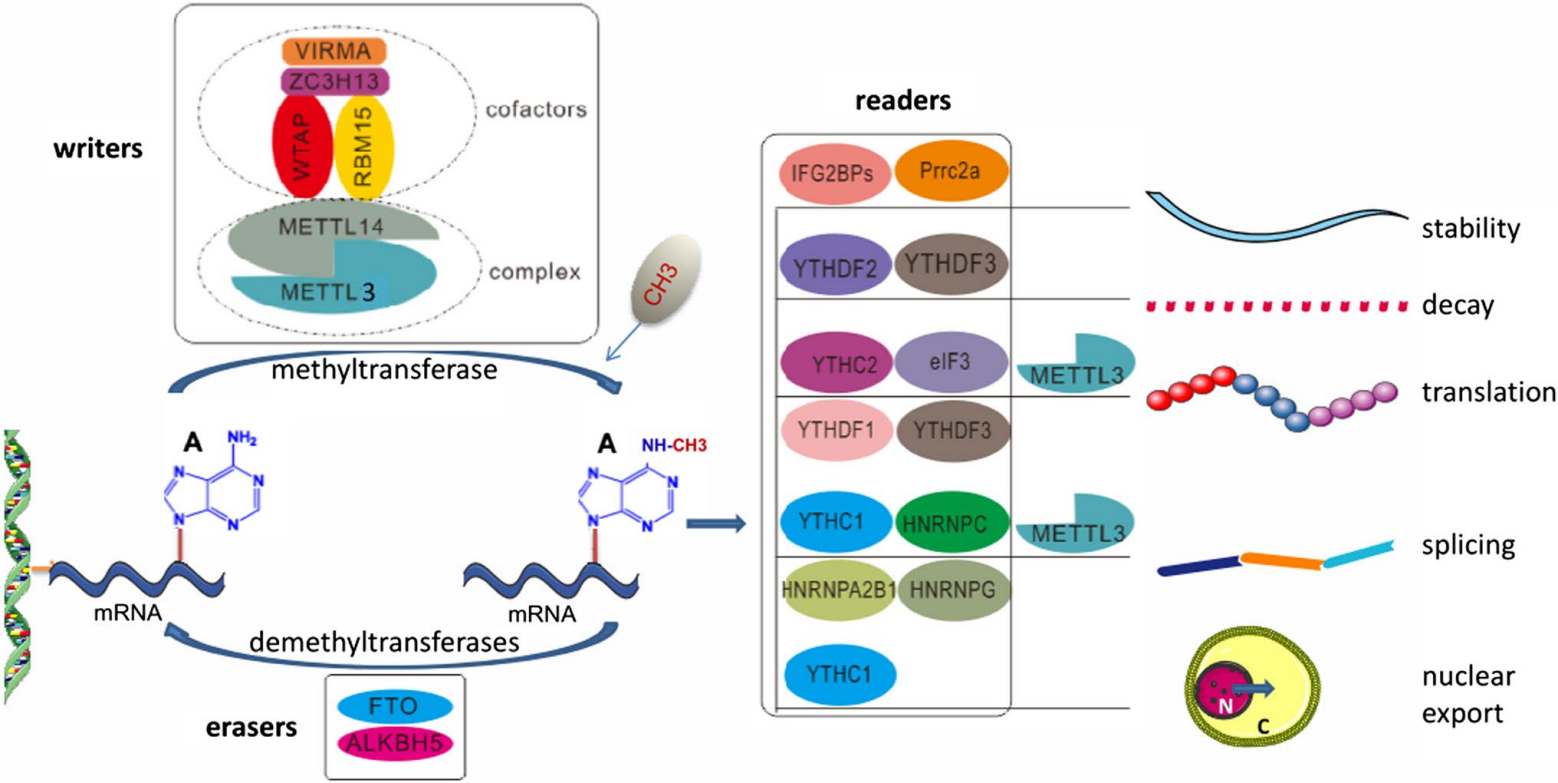
Schematic process and functions of m6A modification. m6A methylation is catalyzed by the writer complex, which includes METTL3, METTL14, WTAP, VIRMA, RBM15, and ZC3H13. The m6A modification is removed by the demethylases FTO or ALKBH5. Readers participate in multiple of steps RNA metabolism, including stability, decay, translation, splicing, and nuclear export

Regulation of m6A is mainly accomplished by three homologous factors, namely the so-called “writers”, “erasers”, and “readers” [11, 22]. The action of m6A itself is catalyzed by the methyltransferase complex (MTC; i.e. the writer enzymes) and removed by demethylases (i.e. the eraser enzymes). The RNA reader proteins, on the other hand, recognize m6A, bind to the RNA, and implement its corresponding translation-related functions (Table 1 and Fig. 1).

**Table 1:** Characteristics and functions of m6A enzymes in RNA metabolism

Category	Gene	Characteristics and functions	References
Writers	METTL3	Promotes formation of the methyltransferase complex, installs m6A on RNA, and catalyzes m6A modification	[14, 22, 25]
	METTL14	Catalyzes the methylation reaction, mediates protein–protein interaction, and helps METTL3 recognize the special RNA substrate	[22, 25]
	METTL16	A homologue of METTL3 Regulator of cellular SAM levels and methylator of U6 small nuclear RNA	[31, 32]
	WTAP	Contributes to the localization of METTL3-METTL14 heterodimer to the nuclear speckle Recruits target RNA for m6A modification Enhances the catalytic capacity of the writer	[26]
	RBM15/15B	Binds the m6A complex and recruit it to special RNA site	[27, 28]
	VIRMA	Recruits the m6A complex to a special RNA site where it interacts with polyadenylation cleavage factors CPSF5 and CPSF6	[30]
	ZC3H13	Bridges WTAP to the mRNA-binding factor to enhance m6A	[29]
Erasers	FTO	Reverses RNA modification and controls cellular homeostasis	[33, 35]
	ALKBH5	Maintains the balance of m6A levels within the transcriptome Contributions to in alkylated DNA repair	[34, 35]
	ALKBH3	Performs its demethylation function on tRNA rather than mRNA or rRNA Generally considered to serve as a DNA repair enzyme	[37]
Readers	YTH family	First five characterized m6A readers	
	YTHDC1	Contributes to RNA splicing and export Plays a critical role in the pre-mRNA processing	[40]
	YTHDC2	Enhances the translation of target RNA but also reduces the abundance of target RNA	[41]
	YTHDF1	Enhances mRNA translation	[43]
	YTHDF2	Promotes mRNA degradation	[44]
	YTHDF3	Enhances translation and degradation by interacting with YTHDF1 and YTHDF2	[45]
	HNRNP family	Another group of RNA-binding proteins	
	HNRNPA2B1	Mediates miRNA processing by recruiting a microprocessor complex upon binding to the m6A	[45, 47]
	HNRNPC/HNRNPG	Mediate selective splicing of m6A-modified transcripts, but do so through direct interaction whit m6A-dependent structural switches	[45]
	eIF3	Initiates the translation procedure by binding to the m6A site in the 5’-UTR of mRNA	[48]
	IGF2BPs	Stabilizes the target gene	[48]
	Prrc2a	Stabilizes mRNA expression by binding to a consensus GGACU motif in the coding sequence in an m6A-dependent manner	[49]

### Methyltransferases/writers

The M6A writers contribute to the MTC formation and include methyltransferase-like 3 and 14 proteins (METTL3 and METTL14) and their cofactors, the WT1-associated protein (WTAP), RNA-binding motif protein 15/15B (RBM15/15B), zinc finger CCCH-type containing 13 (ZC3H13) and Vir-like m6A methyltransferase associated (VIRMA) [11]. METTL3 (also known as MT-A70) was the first component identified in this complex and is the most well understood [23]. It is an S-adenosylmethionine (SAM) binding protein and is highly conserved in various eukaryotic species [24]. It catalyzes the m6A modification and installs m6A on RNA [14, 25]. METTL14 is involved in catalyzing the methylation reaction and mediating protein–protein interaction [22]. In addition, METTL14 supports METTL3 in its recognition of special RNA substrates. WTAP, a splicing factor, is less well understood but studies have indicated a role of mediation in positioning of the heterodimer onto nuclear speckles, as well as for recruitment of the target RNA for m6A modification; the latter would serve to indirectly enhance the catalytic capacity of the writer [26]. Binding of RBM15to the m6A complex facilitates its recruitment to a specific RNA site [27, 28]. ZC3H13 enhances m6A by bridging WTAP to the mRNA-binding factor Nito [29]. VIRMA functions in the 3’ untranslated region (UTR) near the stop codon, directing the m6A complex to a special RNA site where it interacts with the polyadenylation cleavage factors CPSF5 and CPSF6 [30].

In addition to the above-described readers that function in complexes, an independently functioning RNA methyltransferase exists. This homologue of METTL3, termed METTL16, has been characterized as a regulator of cellular SAM levels, a contributor to pre-RNA splicing, and a methylator of U6 small nuclear RNA [31, 32].

### Demethylases/erasers

The dynamic and reversible m6A process also relies on demethylases (erasers). Fat mass and obesity-associated protein (FTO) was the first demethylase discovered, and its ability to reverse RNA modification and control cellular homeostasis is now well characterized. FTO has also been implicated in the adipogenic process, exerting its activity through modulation of the alternative splicing of RUNX1T1 [33]. The AlkB homolog 5 RNA demethylaseALKBH5, expressed primarily in the testes, was the second demethylase identified [34]. The distinct physiological functions of ALKBH5 include regulatory contributions to alkylated DNA repair and maintaining the balance of m6A levels within the transcriptome— the latter being accomplished by working in tandem with FTO [35]. The most recently discovered demethylase, ALKBH3, appears to preferentially demethylate tRNA rather than mRNA or rRNA [36] and is generally considered to serve as a DNA repair enzyme [37].

### Readers

The readers are required for the m6A-modified RNA’s capacity to perform its various biological functions in mRNA translation, degradation, splicing, export, and folding [38]. The first five m6A readers characterized in human represented the YT521-B homology (YTH) domain family [2]. These five proteins, which include two YTHDC members and three YTHDF members, share a conserved m6A-binding domain. YTHDC1 is a nuclear m6A reader, showing almost completely overlapping sites with m6A in nuclear RNAs. Functionally, YTHDC1 contributes to RNA splicing and export, and plays a critical role in the pre-mRNA processing in the oocyte nucleus via interaction with the pre-mRNA3’-end processing factors CPSF6, SRSF3, and SRSF7 [39, 40]. YTHDC2 serves to increase the translation efficiency of target RNA but to also reduce the abundance of target RNA [41]. The three YTHDF proteins recognize the consensus sequence G[G > A]m6ACU [42]. YTHDF1 evokes m6A-containing mRNA translation, upon interaction with an array of translation initiation factors [2, 43]. YTHDF2 harbors a P/Q/N motif with high affinity for mRNAs, resulting in their localization in processing bodies (so-called “P bodies”); the accumulated mRNA undergoes turnover by RNA degradation, a process which involves the YTHDF2 N-terminus and the deadenylase complex CCR4-NOT in mammalian cells [44]. Finally, YTHDF3 enhances RNA translation by interacting with YTHDF1and promotes RNA degradation by associating with YTHDF2 [45].

The heterogeneous nuclear ribonucleoprotein (HNRNP) family of RNA-binding proteins shares a low-complexity domain at the C-terminus. The HNRNPA2B1 member mediates miRNA processing by recruiting a microprocessor complex upon binding to the m6A RGAC motif that is present in a subset of primary miRNA transcripts; this process ultimately facilitates alternative splicing, in a manner similar to that of METTL3 [46, 47]. The HNRNPC and HNRNPG members mediate selective splicing of m6A-modified transcripts, but do so through direct interaction with m6A-dependent structural switches [36]. The translation process itself is then initiated upon the translation initiation factor 3 binding to the m6A site in the 5’-UTR of mRNA, working in conjunction with the insulin-like growth factor 2 mRNA-binding protein family members IGF2BP1/2/3stabilizingthe target gene [48]. The mRNA expression process is further stabilized by interaction between another m6A reader protein, the proline-rich coiled coil 2 A (Prrc2a), which binds to a consensus GGACU motif in the coding sequence [49].

Although each component involved in m6A modification has its own characteristics and functions, they do not function in a mutually exclusive manner. There is much evidence demonstrating collaboration between the writers, erasers, and readers, particularly in the context of cancer pathophysiology [50, 51]. For instance, expression of the YTHDC1 reader shows significant correlation with the expression of several writers, including METTL3 and METTL14; moreover, these correlations are even higher between genes expressing proteins in the same functional complexes, such as RBM15 and WTAP, of the human spliceosome. As these writers, erasers, and readers are known to interact with each other frequently in protein–protein interaction networks, it follows that cross-talk among the enzymes of RNA methylation plays critical roles in the development and progression of various diseases [38].

## Pathophysiological roles of m6A modification in tumor diseases

m6A modification contributes to an array of processes in the formation and progression of cancer, and it can modulate the biological behavior of cancer cells in a variety of ways [52], from cancer stem cell formation and the epithelial–mesenchymal transition to signal transduction and tumor metabolism [17]. Often these, changes occur because of writers adding m6A modifications in the mRNA of oncogenes or tumor suppressor genes and readers that then recognize these biomarkers. Readers can affect the expression of both oncogenes (upregulation) and tumor suppressor genes (downregulation). Conversely, modification removal of the m6A modification by erasers negates the readers’ activities, resulting in the opposite gene regulation effects (oncogene upregulation and tumor suppressor gene downregulation) [53].

Although aberrant m6A modification more commonly contributes to tumorigenesis and tumor progression, recent studies have revealed that abnormal m6A levels can also cause tumor suppression [9]. In glioblastoma stem cells (GSCs), METTL3 promotes mRNA methylation, enhances the stability and expression of SRY-box transcription factor 2 (SOX2), and promotes maintenance and radio-resistance [54]. Additionally, METTL3 has been shown to alter A-to-I and C-to-U RNA-editing events by differentially regulating the RNA-editing enzymes adenosine deaminase acting on RNA and apolipoprotein B mRNA-editing enzyme catalytic subunit 3A. In contrast, some studies have shown that METTL3 is beneficial in the prevention of GSC self-renewal and tumorigenicity [55]. Thus, METTL3 is characterized as a crucial factor throughout the many steps of RNA processing, thereby coordinating the oncogenic pathway in GSCs [56]. In colorectal carcinoma, in particular, METTL3 can facilitate carcinogenesis via an IGF2BP2-dependent process that inhibits SOX2 degradation [57]. In contrast, however, METTL3 interaction with the p38/ERK pathway inhibits the proliferative, migratory, and invasive capacities of colorectal carcinoma cells [58]. In hepatocellular carcinoma (HCC), METTL3-mediated increase in the m6A level of SOCS2 mRNA promotes degradation of this ubiquitous cytokine signaling transducer through an m6A YTHDF2-dependent mechanism; the decreased expression of SOCS2 promotes HCC progression [50]. Simultaneously, YTHDF2 exerts an inhibitory effect on HCC, inhibiting growth of tumor cells and vessels by processing interleukin 11 (IL11) mRNA and serpin family E member 2 (SERPINE2) mRNA [59].

A single m6A modification factor can perform in different ways in different tumors. For example, in lung cancer, YTHDF2 promotes the tumor cell growth by binding directly to the m6A-modified site in the 6-phosphogluconate dehydrogenase (6PGD) 3’-UTR. The resultant increases in translation of the 6PGD mRNA and flux through the pentose phosphate pathway promote the tumor growth [60]. In acute myeloid leukemia, overexpressed YTHDF2 reduces the half-life of tumor necrosis factor receptor superfamily member 2, as well as other m6A-modified transcripts, supporting the leukemic stem cell niche [61]. In breast cancer, a YTHDF2-dependent mechanism underlies the degradation of the BCL2 interacting protein 3 (BNIP3) mRNA, which would otherwise encode an apoptosis-promoting protein that is a downstream target of FTO-mediated m6A modifications [62]. YTHDF2 can also inhibit HCC tumor cells and related vessels by processing IL11 and SERPINE2 mRNAs [59].

## Therapeutic applications

A growing number of studies are revealing many potential therapeutic applications of m6A for clinical use. For example, (i) tumor neoantigens generate spontaneous antitumor immunity and can serve as biomarkers for evaluating clinical responses to immunotherapies [63]. A recent study indicated that YTHDF1 can suppress the cross-priming ability of dendritic cells. Loss of YTHDF1 then promotes infiltration of neo-antigen-specific CD8^+^ T cells and enhances the cross-presentation of tumor antigens to inhibit tumors growth. Furthermore, given that YTHDF1 depletion promotes interferon gamma (IFN-γ) production, followed by an increase in PD-L1 in CD8^+^ T cells [64], it follows that combining a PD-L1 checkpoint inhibitor with YTHDF1 depletion would produce a stronger therapeutic efficacy. In combination with the more recent checkpoint blockade strategy, YTHDF1 could be a potential new therapeutic target for immunotherapy. (ii) FTO, which had previously only been studied as an inhibitor in tumors, was found to be higher in cervical squamous cell carcinoma tissue than in the corresponding para-cancerous tissues. FTO increases the expression of excision repair cross-complementation group, thereby contributing to chemo- and radio-resistance and showing potential clinical significance for the treatment of this cancer type [65, 66]. (iii) In a mouse model of leukemia, cells with mRNA m6A hypo-methylation and FTO upregulation was found to promote tolerance to tyrosine kinase inhibitor treatment and growth rates. FTO deactivation (m6A methylation inducement by genetic or pharmacologic means) restores the sensitivity to tyrosine kinase inhibitors [66]. (iv) Chen et al. [67] showed that m6A RNA methylation regulators are closely related to malignant clinico-pathological features of breast cancer and identified a prognostic bio-signature, composed of the FTO, YTHDC1 and WTAP m6A RNA methylation regulators. We speculate that similar studies will highlight the therapeutic applicability of different m6A RNA methylation regulators to other tumors [68].

## m6A involvement in melanoma

**Fig. 2:**
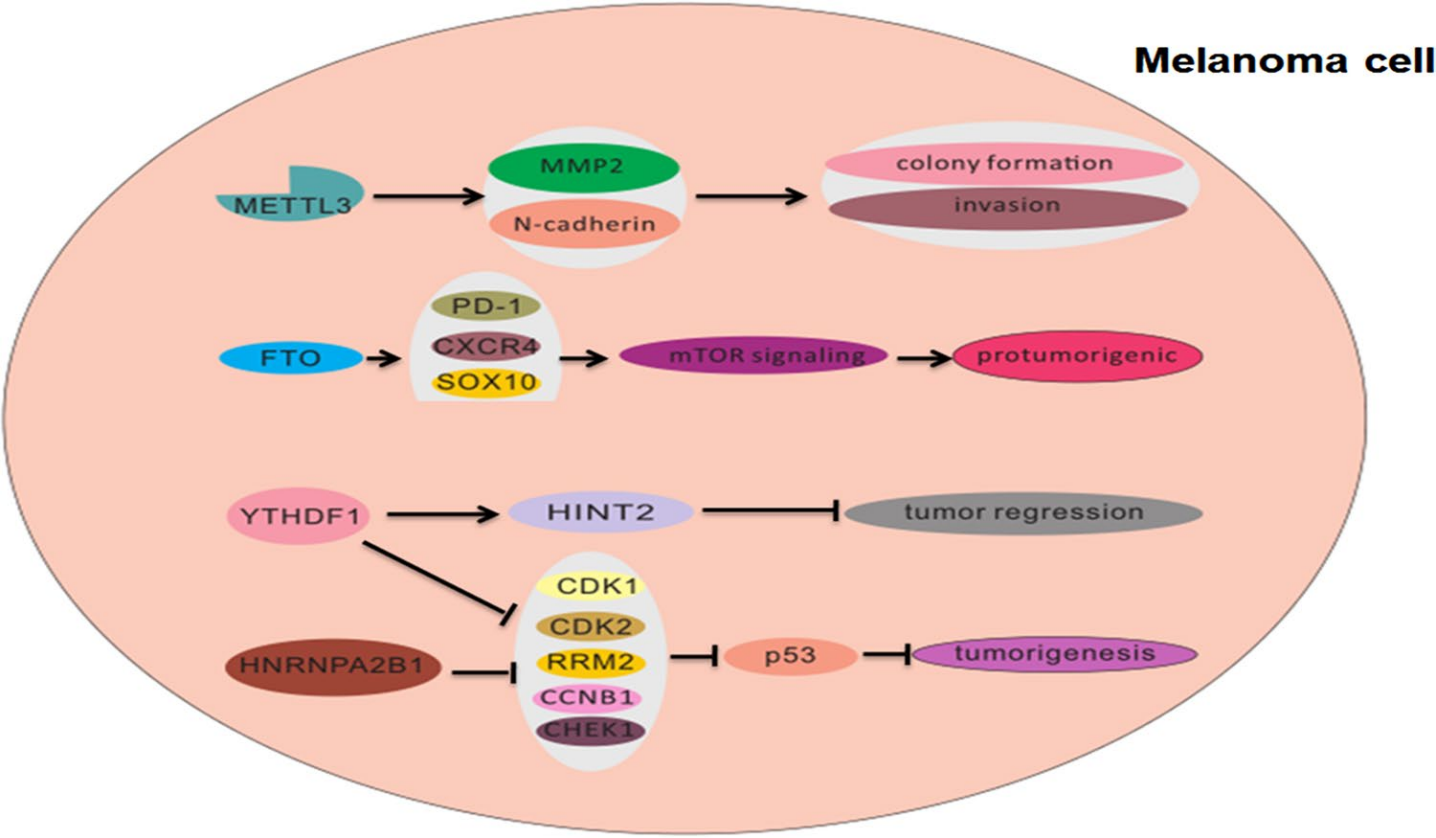
Potential roles of m6A modification in melanoma pathogenesis. The effect of m6A on melanoma cells is reflected in the regulation of cancer-related gene expression

Melanoma is the fifth-most common cause of cancer overall in the United States [69]. Between 2006 and 2015, the overall incidence rate of melanoma increased from 200.1 to 229.1 cases per million person-years and the death rate is more than 9000 per year [70]. Although progress has been made in the treatment of advanced melanoma [71, 72], there remain no proper curative treatments currently available. Melanoma is a type of epithelial malignant tumor that originates from melanocytes [73]. Advances in sequencing technology have led to the identification of pathogenic mutations in the B-Raf proto-oncogene (BRAF) and related signaling pathways, such as the mitogen-activated protein kinase (MAPK) pathway and phosphatidylinositol 3-kinase-AKT pathway [74, 75]. The BRAF mutations are associated with decreased disease-free and melanoma-specific survival. These discoveries have led to the emergence of targeted drug treatments for melanoma, such as BRAF inhibitors, MAP2K7 (also known as MEK), and MAPK1 (also known as ERK) [76, 77]. The Telomerase Reverse Transcriptase (hTERT) promoter mutations, which were first identified in melanomas, often in combination with BRAF mutations, were reported to activate the telomerase and to be implicated in tumorigenesis [78, 79]. Also, some studies linked these mutations to the treatment efficacy or resistance [79, 80]. However, the actual pathological mechanism of melanoma remains unknown. Considering that m6A methylation is closely related to tumorigenesis and tumor development [65, 81], several studies have investigated the roles for such in the pathological processes of melanoma (Fig. 2).

Though many cancers show deregulated expression of m6A and METTL3, the role of each in melanoma still remains to be fully elucidated. Using in vitro systems, Dahal et al. [82] found that melanoma cell lines express higher levels of METTL3 than normal melanocytes. Silencing of the METTL3 gene expression reduced not only the m6A activity but also the cells’ capacities for colony formation and invasiveness, with over-expression of the METTL3 gene producing the opposite effects upon accumulation of MMP2 and N-cadherin. Overexpression of m6A catalytic site mutant in METTL3 did not produce a similar increase in MMP2, suggesting that m6A activity of METTL3 is important for melanoma cell invasiveness [83]. Though the results need to be confirmed by in vivo studies, they suggest a potential therapeutic benefit of METTL3 inhibitors in melanoma.

**Fig. 3:**
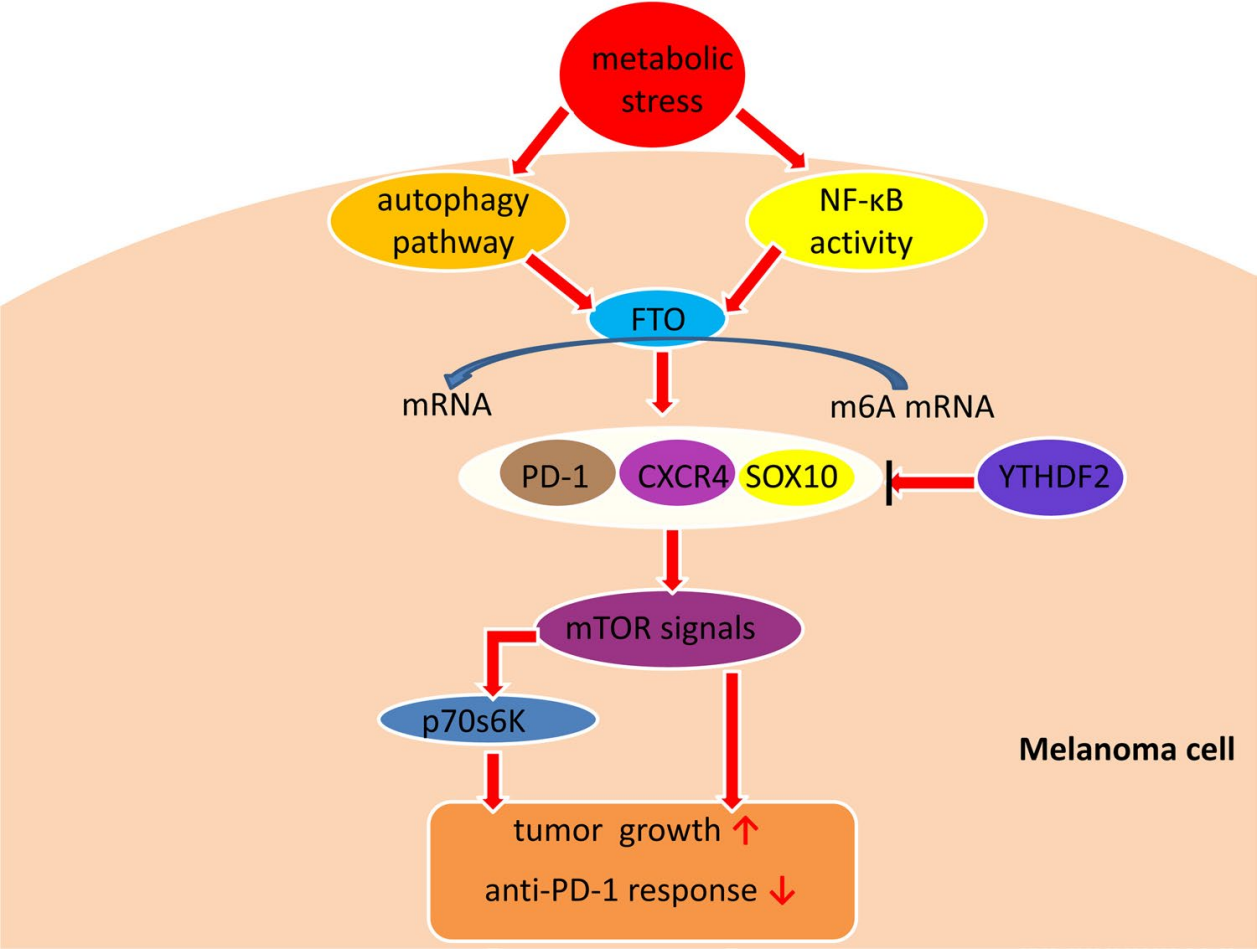
Mechanistic illustrations of the regulatory and functional roles for FTO in melanoma. FTO can regulate cell-intrinsic expressions of PD-1, CXCR4, and SOX10 to promote the growth and anti-PD-1 response of melanoma

Research on FTO has suggested that m6A RNA methylation is reversible and dynamic and may have crucial physiological and pathological functions [84]. Human melanoma tissues show upregulated FTO [85]. FTO expression is regulated by autophagy pathway and nuclear factor kappa B pathways and can be induced by metabolic stress; the FTO protein itself regulates the expression of known melanoma-related genes encoding PD-1 (PDCD1), CXCR4, and SOX10. Indeed, knockdown of FTO reduced the levels of these proteins but also had a negative impact on the phosphorylation of p70s6K, a substrate of mTOR activation [85]. PD-1 activates pathways downstream of mTOR, itself a tumorigenic factor that promotes melanoma tumor growth [86]. Increasing the general knowledge on the regulatory mechanisms of PD-1 under normal physiologic conditions may help us to better understand its regulatory roles in cancer biology and immunotherapy response in melanoma; indeed, one study has already used PD-1 to successfully target immune cells as a cancer immunotherapy [87]. The collective results from these PD-1 studies have indicated the critical role of melanoma cell-intrinsic FTO in promoting melanoma resistance, such as to anti-PD-1 blockade [85] (Fig. 3). Ultimately, however, they suggest that the regulation of these melanoma-promoting genes by FTO and other proteins might critically affect gene expression in melanoma (*e.g*., the methyltransferases METTL3 and METTL14) and further studies on the therapeutic potential of such are warranted.

As described above, the regulation of gene expression by m6A is executed mostly through the readers, including YTH domain-containing family proteins in mammalian cells. Yang and colleagues [85] found that knocking down only YTHDF2 significantly increased the mRNA stability of PD-1 (PDCD1), CXCR4, and SOX10; in conjunction, the proliferative and migratory capacities of the melanoma cells were increased (in vitro) as was tumor growth (in vivo). In melanoma cells, YTHDF2 would serve as a tumor suppressor, mediating the downregulation of the FTO target genes. Although in normal melanoma, the other family member YTHDF1 does not reduce cell proliferation and migration, it can promote translation of the histidine triad nucleotide-binding protein 2 tumor suppressor in ocular melanoma, being shown to promote tumor regression in vitro and in vivo [88].

One systematic analysis revealed that the expressions of YTHDF1 and HNRNPA2B1 were upregulated in melanoma [89]. Expression screening for both of these genes, in combination, was also found to improve melanoma diagnosis rates by about 10% (versus mono-gene screening). Furthermore, the study found that genes related to p53-signaling (namely, CDK2, CDK1, RRM2, CCNB1, and CHEK1) positively correlated with the observed YTHDF1 or HNRNPA2B1 upregulation. Thus, both genes may affect m6A modification of the identified five genes in particular, promoting the ability of the encoded proteins to inhibit p53 and suppress tumorigenesis. Finally, the study found that upregulating mutations in YTHDF1 and HNRNPA2B1 correlated with tumor stage and treatment response in patients, further supporting their roles, asm6A regulatory genes, in melanoma.

m6A modification contributes to an array of processes in the formation and progression of cancer. In malignant ocular melanoma, the “writer” METTL3, lower expression predicted earlier recurrence and enhanced aggressiveness, significantly decreased in m6A modification in cellular mRNAs, while the “eraser” ALKBH5, the expression indicated a poor prognosis, was observed the opposite trend. This study indicates that the disturbance of global m6A homeostasis, which depends on the balance of “writers” and “erasers” of m6A modification, is a key driver in the regulation of tumor formation [88].

In short, these studies on m6A in melanoma have advanced our understanding of the pathogenesis and biological characteristics of melanoma, in general, and may provide foundational data to improve future diagnosis and development of molecular targeted therapy and immunotherapies. However, research on the role of m6a in melanoma is relatively limited, additional research is necessary to elucidate the molecular mechanisms of m6A in melanoma.

## Conclusion and perspectives

The m6A modification of RNA is becoming recognized as an important post-transcriptional regulation of gene expression, both under physiologic and pathogenic conditions. Deregulation of the underlying m6A regulators affects a vast array of downstream targets, disrupting mRNA stability and impeding translation efficiency. The implications of m6A modification in human carcinogenesis have been demonstrated in different cancer types, including melanoma. However, the mechanisms underlying m6A modification and m6A regulators are heterogeneous and complex. Identification of cancer-specific m6A modifications will be important, as they may serve as potential diagnostic and prognostic markers or as targets for development of novel therapies.

While the effects of m6A modification are known for many types of tumors, effects on non-tumor diseases and physiological processes are only beginning to be recognized. Many of the m6A transcripts encoded by viruses have been identified [90] and their functions in viral–host interactions are believed to be important in human disease. For example, m6A modifications to RNAs of the human respiratory syncytial virus (RSV) enhance viral replication and pathogenesis; results from knockdown studies of the m6A methyltransferases and demethylases have suggested that viral m6A methylation could be a therapeutic target [91]. In addition, under stress conditions, mRNA methylation in human and mouse cardiomyocytes is highly dynamic; the mRNA methylome may be a manipulable regulator of translational efficiency, based upon its effects on transcript stability [92]. Once more m6A methylation mechanisms are elucidated, targeted molecular manipulation could be a powerful approach to preventive care, such as worsening of cardiac stress.

Interestingly, m6A modification has also been implicated in homeostasis of neuronal and immune systems. Chen and colleagues [93] found that depletion of METTL3, in particular, significantly reduced m6A levels in adult neural stem cells (aNSCs) and inhibited their proliferation. Ultimately, neuronal development was inhibited and differentiation of the aNSCs became skewed towards a glial lineage, effects that were rescued by Ezh2 over-expression [93]. These results may provide insight into central nervous system diseases. M6A modification was also recently characterized as a crucial regulator of T cell homeostasis and the immune response to bacterial or viral infection [31], highlighting an immunotherapeutic potential for such.

So far, m6A modification has been found to play significant roles in cancers and it can provide a series of new pharmacological targets. Taketo K et al. show that the METTL3-depleted have higher sensitivity to anti-cancer reagents, such as gemcitabine, 5-fluorouracil, cisplatin and irradiation, than those without METTL3-depleted in pancreatic cancer patients [94]. Whether the same results will apply to melanoma remains unknown, thus further studies should be performed to understand m6A modification in melanoma and other cancers and more highly selective and potent inhibitors of m6A modification should be required. It is well known that immunotherapy plays an important role in the treatment of melanoma, a variety of researches have confirmed RNA m6A methylation can modulate innate/adaptive immunity responses [95]. For instance, T cells modified to carry m^6^A-modifying agents could be an efficient therapeutic strategy for various autoimmune diseases [96], which also suggests the potential application of such strategy in cancer immunotherapy. Although these results have not yet been verified in vivo and in clinical trials, it can provide potential pharmacological targets for anti-cancer drug development and cancer immunotherapy for melanoma.
